# Vaccine targeting HIF1A in triple negative breast cancer

**DOI:** 10.1186/2051-1426-2-S3-O5

**Published:** 2014-11-06

**Authors:** Denise Cecil, Daniel Herendeen, Meredith Slota, Yushe Dang, Megan O'Meara, Ekram Gad, Lauren Rastetter, Marlese Koehnlein, Mary L Disis

**Affiliations:** 1 University of Washington, Seattle, WA, United States; 2 Seattle Genetics, Seattle, WA, United States

## 

The high rates of relapse in triple negative breast cancer (TNBC) are thought to be due to the presence of increased levels of cancer stem cells (CSC), which have been shown to be resistant to standard therapies. It has been demonstrated that hypoxia-inducible factor 1 (HIF1A) can induce the expression of numerous gene products associated with stem-ness and epithelial-mesenchymal transition in breast cancer cells and has been shown to be hyperactivated in TNBC. In this study, we aimed to target HIF1A with a therapeutic immune response through active immunization. HIF1A is a tumor-associated antigen. We have determined that both the magnitude and incidence of HIF1A-specific IgG is significantly elevated in TNBC compared to volunteer donors. We identified epitopes derived from HIF1A that selectively elicited IFN-gamma secretion with little to no IL-10 secretion in human peripheral blood mononuclear cells and T cell lines generated with these epitopes responded to recombinant HIF1A protein. Furthermore, these epitopes are highly homologous between mouse and man. To evaluate therapeutic efficacy, we immunized MMTV-neu (HER2^+ ^model) and C3(1)Tag (TNBC model) mice with a plasmid-based vaccine containing an extended sequence of the identified epitopes. Tumor growth was inhibited over 80% (p < 0.0001) in the TNBC model; however, growth was inhibited only by 40% (p < 0.01) in the HER2^+ ^model. We determined the majority of the tumor cells from the TNBC model expressed the mouse stem cell marker, Sca-1, whereas only a minority of the cells derived from the HER2^+ ^model expressed the marker. Finally, we detected a 52% decrease in tumor Sca-1 expression after HIF1A-specific vaccination in the TNBC model (p = 0.004). Targeting HIF1A via active immunization may be an effective way to prevent disease relapse in patients with TNBC.

